# Risk factors for poor prognosis in neonatal sepsis: a systematic review and meta-analysis

**DOI:** 10.3389/fmed.2026.1979094

**Published:** 2026-10-14

**Authors:** Dejuan Yang, Yan Han, Liping Wang, Wenhua Lü

**Affiliations:** 1 Department of Laboratory Medicine, Qinghai Provincial Women and Children's Hospital, Xining, Qinghai, China; 2 Neonatal Mother–Infant Rooming-in Unit, Qinghai Provincial Women and Children's Hospital, Xining, Qinghai, China; 3 Department of Neonatology, Qinghai Provincial Women and Children's Hospital, Xining, Qinghai, China

**Keywords:** meta-analysis, neonate, poor prognosis, risk factors, sepsis, systematic review

## Abstract

**Objective:**

To synthesize previous research findings through systematic review and meta-analysis and to evaluate risk factors associated with poor prognosis in neonatal sepsis, thereby providing additional evidence for early clinical risk assessment.

**Methods:**

According to the study objectives, population, and study design, the inclusion criteria, exclusion criteria, and detailed search strategy were established. Eight databases were searched, including PubMed, Embase, Web of Science, the Cochrane Library, China National Knowledge Infrastructure (CNKI), Wanfang Data, VIP Chinese Science and Technology Journals Database, and the Chinese Biomedical Literature Database (CBM), from database inception to January 21, 2026. The specific coverage periods were as follows: PubMed (1946 to January 21, 2026), Embase (1947 to January 21, 2026), the Cochrane Library (1992 to January 21, 2026), Web of Science (1900 to January 21, 2026), CNKI (1915 to January 21, 2026), VIP Chinese Science and Technology Journals Database (1989 to January 21, 2026), CBM (1994 to January 21, 2026), and Wanfang Data (1998 to January 21, 2026). Studies meeting the inclusion criteria were selected. Study selection and data extraction were independently performed by two reviewers. Methodological quality was assessed using the Newcastle-Ottawa Scale, and statistical analyses were conducted using RevMan 5.4.

**Results:**

A total of 26 studies were included. The main independent risk factors for poor prognosis in neonatal sepsis were as follows: ① elevated interleukin-6 [*OR* = 1.97, *p* < 0.001]; ② elevated procalcitonin [*OR* = 1.59, *p* < 0.001]; ③ thrombocytopenia [*OR* = 2.47, *p* = 0.020]; ④ elevated C-reactive protein [*OR* = 2.04, *p* = 0.003]; ⑤ leukopenia [*OR* = 2.90, *p* = 0.001]; ⑥ elevated lactate [*OR* = 2.18, *p* = 0.005]; ⑦ Gram-negative bacterial infection [*OR* = 5.37, *p* = 0.002]; and ⑧ decreased albumin [*OR* = 5.77, *p* = 0.001].

**Conclusion:**

Elevated inflammatory biomarkers, hematologic abnormalities, hyperlactatemia, hypoalbuminemia, and Gram-negative bacterial infection are important indicators of poor prognosis in neonatal sepsis. Early recognition of these factors and timely intervention may improve clinical outcomes.

**Systematic Review Registration:**

https://www.crd.york.ac.uk/PROSPERO/view/CRD420261290404, CRD420261290404.

## 1 Introduction

Neonatal sepsis (NS) is an infectious disease in which pathogen invasion triggers a severe systemic inflammatory response (1). Clinical manifestations include poor general condition, respiratory distress, marked temperature instability, tachycardia, feeding intolerance, jaundice, and shock (2). Neonatal sepsis accounts for approximately 15% of neonatal deaths worldwide, and the case-fatality rate in China is about 13.0% (3). Because early manifestations are often subtle, delayed recognition and treatment may result in severe complications, create major diagnostic and therapeutic challenges, and threaten the infant's life (4). The disease also imposes a substantial public-health and socioeconomic burden worldwide (5). Antibiotics remain the cornerstone of neonatal sepsis treatment (6). Immunoglobulin combined with meropenem has shown potential benefits by attenuating cellular inflammatory responses and improving immune function; however, the clinical evidence supporting this approach requires further verification (7). Prevention and effective treatment of neonatal sepsis are therefore crucial for improving neonatal health and survival (8). Most existing studies have focused on diagnosis and acute-phase treatment, whereas risk factors for poor prognosis have not been comprehensively identified. Early warning strategies and individualized measures for improving outcomes in high-risk neonates also remain insufficient, and systematic intervention programs are lacking. Accordingly, this study systematically searched the Chinese and international literature on risk factors for poor prognosis in neonatal sepsis and synthesized the available evidence through meta-analysis to provide a scientific basis for clinical prognostic assessment.

## 2 Methods

### 2.1 Eligibility criteria

Studies were included when they met all of the following criteria: (1) participants were neonates who met diagnostic criteria for neonatal sepsis (9), were aged 28 days or younger, were clinically stable, and had no hematologic disease, malignant tumor, pulmonary tuberculosis, or other chronic wasting disease; (2) studies reported laboratory indicators or other risk factors potentially affecting prognosis, with poor prognosis defined as death, death after withdrawal of treatment, or severe neurologic sequelae; and (3) the study was a cohort or case-control study that reported complete effect estimates, such as odds ratios (ORs), hazard ratios (HRs), and 95% confidence intervals (CIs), or provided raw data from which these measures could be calculated.

### 2.2 Exclusion criteria

(1) neonates with severe congenital malformations, inherited metabolic disorders, other serious diseases, or infectious diseases; (2) studies that did not report 95% confidence intervals (CIs), did not provide sufficient data to extract effect estimates suitable for meta-analysis, used outcome definitions inconsistent with those prespecified in this review, had unavailable full texts, or contained missing or erroneous data; (3) duplicate publications; (4) studies published in languages other than Chinese or English; and (5) conference papers, case reports, reviews, and animal studies.

### 2.3 Search strategy

This study was conducted and reported in strict accordance with the Preferred Reporting Items for Systematic Reviews and Meta-Analyses (PRISMA) guidelines. The registration status, search strategy, eligibility criteria, and synthesis methods were as follows. The review was prospectively registered on October 7, 2025, under registration number CRD420261290404. A comprehensive computerized search was conducted in PubMed, Web of Science, the Cochrane Library, Embase, the VIP Chinese Science and Technology Journals Database, China National Knowledge Infrastructure (CNKI), the Chinese Biomedical Literature Database (CBM), and Wanfang Data to identify studies investigating factors associated with poor prognosis in neonatal sepsis. Each database was searched from its inception to January 21, 2026. Controlled vocabulary terms and free-text terms were combined using the Boolean operators AND and OR. All retrieved records were imported into EndNote X9 for deduplication, and the study selection process was documented using a PRISMA flow diagram. Eligibility criteria were predefined according to the PICOS framework. Based on the prespecified inclusion and exclusion criteria, the basic characteristics of eligible studies were extracted and their methodological quality was assessed. Quantitative synthesis was performed using RevMan 5.4, including assessment of between-study heterogeneity and potential publication bias where appropriate. The search terms included “Neonatal Sepsis,” “Neonatal Bacteremia,” “Sepsis Neonatorum,” “Poor Prognosis,” “Outcome,” “Risk Factors,” “Influencing Factors,” and “Predictors.” The PubMed search strategy is presented in Box 1-1.

Box 1-1. PubMed search strategy.

**Table:** 

Database	Search date	string		Number of hits	Time
PubMed	2026-01-21	10	#3 AND #6 AND #9	185	01:04:41
		9	#7 OR #8	1,312,537	01:04:22
		8	"Factor,Risk"[Title/Abstract] OR “Risk Factor"[Title/Abstract] OR “Population at Risk"[Title/Abstract] OR “Populations at Risk"[Title/Abstract] OR “Risk Scores"[Title/Abstract] OR “Risk Score"[Title/Abstract] OR “Score, Risk"[Title/Abstract] OR “Risk Factor Scores"[Title/Abstract] OR “Risk Factor Score"[Title/Abstract] OR “Score, Risk Factor"[Title/Abstract] OR “Health Correlates"[Title/Abstract] OR “Correlates, Health"[Title/Abstract] OR “Social Risk Factors"[Title/Abstract] OR “Factor, Social Risk"[Title/Abstract] OR “Factors, Social Risk"[Title/Abstract] OR “Risk Factor, Social"[Title/Abstract] OR “Risk Factors, Social"[Title/Abstract] OR “Social Risk Factor"[Title/Abstract]	382,308	01:03:54
		7	"Risk Factors"[Mesh]	1,084,470	01:01:38
		6	#4 OR #5	3,959,153	01:01:04
		5	"mortality"[Title/Abstract] OR “survival"[Title/Abstract] OR “overall survival"[Title/Abstract] OR “disease-specific survival"[Title/Abstract] OR “prognosis"[Title/Abstract] OR “disease progression"[Title/Abstract] OR “disease recurrence"[Title/Abstract] OR “recurrence"[Title/Abstract] OR “relapse"[Title/Abstract] OR “neoplasm metastasis"[Title/Abstract] OR “metastasis"[Title/Abstract] OR “treatment failure"[Title/Abstract] OR “drug resistance"[Title/Abstract] OR “chemotherapy resistance"[Title/Abstract] OR “refractory"[Title/Abstract] OR “adverse outcomes"[Title/Abstract] OR “unfavorable outcome"[Title/Abstract] OR “poor outcome"[Title/Abstract]	3,881,819	01:00:20
		4	"Mortality"[Mesh]	452,341	00:59:34
		3	#1 OR #2	2,119	00:58:10
		2	(“Neonatal Sepses"[Title/Abstract] OR “Sepses, Neonatal"[Title/Abstract] OR “Sepsis, Neonatal"[Title/Abstract] OR “Neonatal Early-Onset Sepsis"[Title/Abstract] OR “Early-Onset Sepses, Neonatal"[Title/Abstract] OR “Early-Onset Sepsis, Neonatal"[Title/Abstract] OR “Neonatal Early-Onset Sepses"[Title/Abstract] OR “Neonatal Early Onset Sepsis"[Title/Abstract] OR “Sepses, Neonatal Early-Onset"[Title/Abstract] OR “Sepsis, Neonatal Early-Onset"[Title/Abstract] OR “Neonatal Late-Onset Sepsis"[Title/Abstract] OR “Late-Onset Sepses, Neonatal"[Title/Abstract] OR “Late-Onset Sepsis, Neonatal"[Title/Abstract] OR “Neonatal Late-Onset Sepses"[Title/Abstract] OR “Neonatal Late Onset Sepsis"[Title/Abstract] OR “Sepses, Neonatal Late-Onset"[Title/Abstract] OR “Sepsis, Neonatal Late-Onset"[Title/Abstract])	338	00:57:22
		1	"Neonatal Sepsis"[Mesh]	1,932	23:49:56

### 2.4 Study selection and data extraction

After preliminary deduplication in EndNote X9, two reviewers independently screened titles and abstracts. Potentially eligible records underwent full-text assessment. Study selection, data extraction, and cross-checking were performed independently, and disagreements were resolved through discussion and consensus.

### 2.5 Quality assessment

Two trained reviewers independently assessed methodological quality using the Newcastle-Ottawa Scale (NOS). The NOS evaluates selection of participants, comparability between groups, and outcome assessment, with a maximum score of 9. Studies scoring 7-9 were considered high quality, those scoring 4–6 were considered moderate quality, and those scoring below 4 were considered low quality (10).

### 2.6 Statistical analysis

RevMan 5.4 was used to analyze the risk factors reported in the included studies. Heterogeneity was evaluated using the *I^2^* statistic. When *p* ≥ 0.10 and *I^2^* ≤ 50%, heterogeneity was considered low and a fixed-effects model was applied; when *p* < 0.10 or *I^2^* > 50%, a random-effects model was used. Studies that could not be quantitatively pooled were summarized descriptively. Statistical significance was set at *p* < 0.05. For dichotomous variables, ORs and corresponding 95% CIs were used as effect measures. Sensitivity analyses were conducted using a leave-one-out approach, in which each study was sequentially excluded and the pooled estimate recalculated to explore potential sources of heterogeneity.

## 3 Results

### 3.1 Study selection

The database search identified 7,117 records: 185 from PubMed, 1,318 from Embase, 472 from the Cochrane Library, 1,582 from Web of Science, 280 from Wanfang Data, 29 from VIP, 3,236 from CNKI, and 15 from CBM. After 820 duplicates were removed, 6,297 records remained. Title and abstract screening excluded 5,380 records, and 917 full-text articles were assessed. Of these, 605 had ineligible populations, 263 had inconsistent outcome measures, 6 full texts were unavailable, 15 were cross-sectional studies, and 2 were randomized controlled trials. Ultimately, 26 studies, including 18 cohort studies and 8 case-control studies, were included in the meta-analysis (Figure 1).

**Figure 1:**
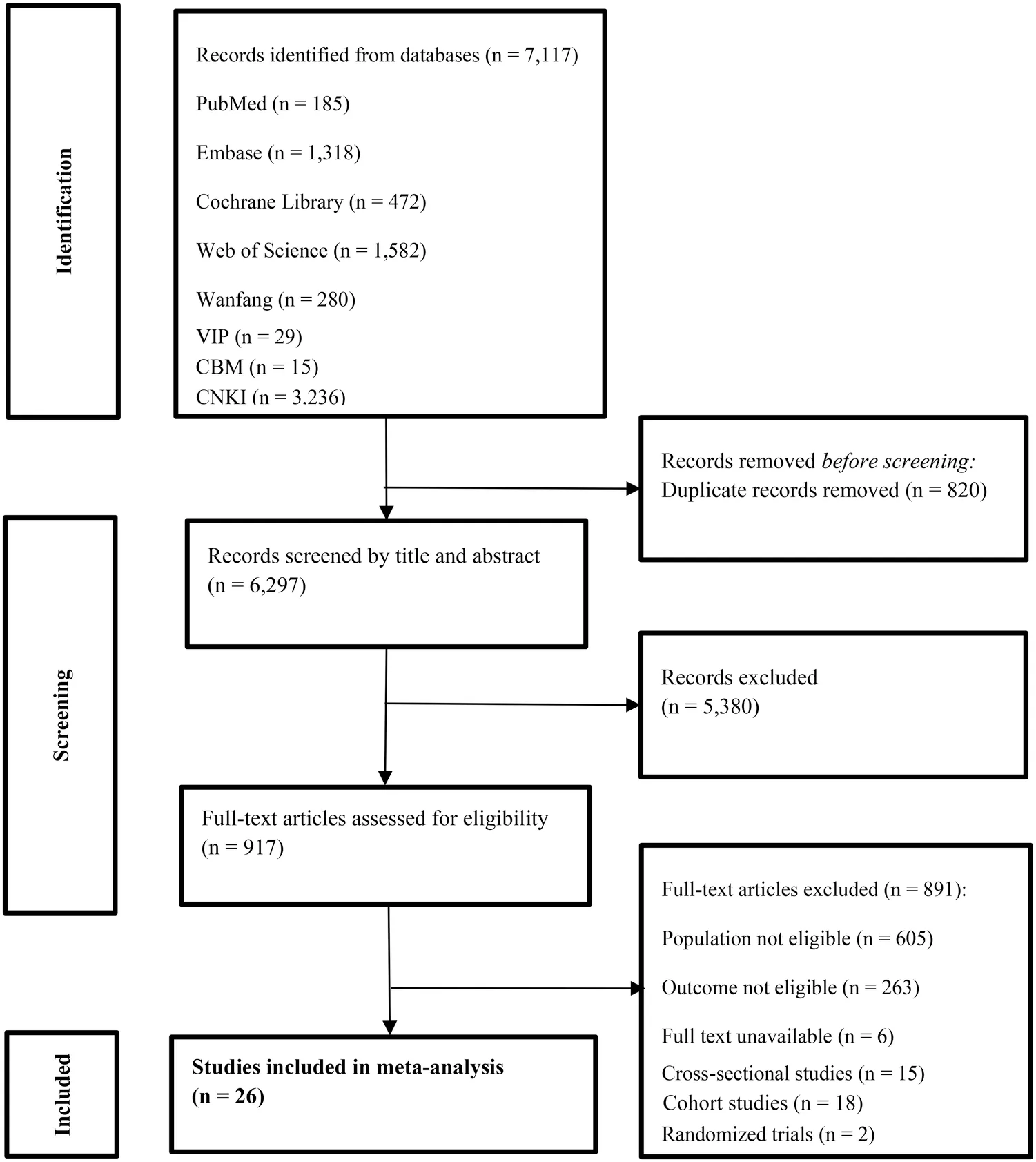
Flow diagram of study selection.

### 3.2 Study characteristics and quality assessment

Twenty-six studies were included. Extracted characteristics comprised first author, country, publication year, sample size, study period, risk factors, outcomes, effect measure (*OR* or *HR*), and 95% *CI*. The studies were cohort or case-control studies conducted in hospitals, with sample sizes ranging from 60 to 3,862 participants. Fifteen studies were conducted in China, two in the Netherlands, and one each in Vietnam, the United States, Korea, India, Egypt, and Thailand. According to the NOS, all studies were of moderate or high methodological quality. Detailed characteristics are shown in Table 1.

**Table 1:** Characteristics and quality assessment of the included studies.

First author	Country	Sample size	Study design	Adjusted covariates	Cut-off value	Prognostic factors	Outcome	Effect measure	NOS score
Jia Panqiang 2025 (11)	China	2,412	Cohort	Disease severity; type of sepsis	① 4.17 μg/mL; ② 1.73 ng/mL; ③ 1.51 pg/mL	①②③	Composite	*OR*	9
Fan Lili 2023 (12)	China	72	Cohort	Premature rupture of membranes >12 h	None	④	Composite	*OR*	7
Xu Aihua 2024 (13)	China	86	Cohort	Score on the neuropsychological development assessment for children aged 0–6 years	Not reported	⑤⑥⑦	Mortality	*OR*	7
Hu Yurun 2025 (14)	China	84	Cohort	PCT	⑧ > 19.68 ng/mL; ⑨ > 2.91 ng/L	⑧⑨	Composite	*OR*	8
Yu Dongmei 2012 (15)	China	87	Cohort	Shock; MODS; PCIS	Not reported	⑩	Mortality	*OR*	8
Kurul 2021 (16)	Netherlands	143	Cohort	Previous sepsis	Not reported	⑤⑥	Mortality	*HR*	6
Toan 2022 (17)	Vietnam	524	Cohort	Sclerema neonatorum; very low birth weight	Not reported	⑩⑪⑫⑬⑭	Mortality	*OR*	6
Levit 2014 (18)	United States	424	Cohort	Adjusted covariates were not reported in publicly available materials; supplementary data were unavailable	None	⑮⑯⑰	Mortality	*OR*	6
Kim 2018 (19)	Korea	3,862	Cohort	Gestational age; birth weight	Not reported	⑱⑲	Mortality	*OR*	7
Wang 2025 (20)	China	69	Cohort	Age; sex; underlying diseases	Not reported	⑳	Mortality	*HR*	6
Xia 2022 (21)	China	134	Cohort	Age; white blood cell count; lymphocyte count; monocyte count	Not reported	㉑	Mortality	*HR*	7
Xia 2025 (22)	China	130	Cohort	Platelet count; AST; LDH; albumin/globulin ratio; creatinine	㉒ 28.4 g/L; ㊷ 23.72	㉒㊷	Mortality	*HR*	8
Ree 2017 (23)	Netherlands	460	Cohort	Small for gestational age (SGA); cesarean delivery; maternal hypertension; gestational age ≤32 weeks; intravascular thrombosis; Gram-negative sepsis	Not reported	⑩	Mortality	*OR*	7
Zeng Zhen 2024 (24)	China	60	Cohort	Gestational age; birth weight; electrolyte and acid–base disturbances; pathological jaundice; mechanical ventilation; poor responsiveness; septic shock; Hb	Not reported	⑩⑪	Composite	*OR*	7
Zhan Yuanli 2021 (25)	China	538	Cohort	Gestational age; septic shock; necrotizing enterocolitis (NEC)	Not reported	㉓	Mortality	*OR*	6
Zhang Ting 2023 (26)	China	99	Cohort	None	Not reported	㉔㉕㉖	Composite	*OR*	7
Tu 2025 (27)	China	195	Cohort	Platelet count; ALT; creatinine; PT; INR; albumin; TT	Not reported	㉗㉘㉙	Mortality	*HR*	8
Liu 2020 (28)	China	472	Cohort	Total calcium <1.8 mmol/L	Not reported	㉒㉚㉛㉜	Mortality	*OR*	8
Chen Yubing 2025 (29)	China	65	Case-control	Model B: change in feeding behavior (for the IL-6 estimate); Model C: change in feeding behavior and jaundice (for the IL-8 estimate)	Not reported	⑤㉝	Mortality	*OR*	6
Salim 2020 (30)	Egypt	100	Case-control	SNAP-II score; PCT	㉞ 3.48	㉞	Mortality	*OR*	8
Sahu 2022 (31)	India	4,690	Case-control	Mechanical ventilation; continuous positive airway pressure (CPAP); peripheral venous catheterization; pulmonary surfactant therapy; surgical procedures; blood transfusion	Not reported	⑦⑩⑪㉟	Mortality	*OR*	7
Zhu Hongfen 2021 (32)	China	256	Case-control	Very low birth weight; history of PICC placement; Apgar score <7; intrauterine growth restriction	⑤ 148.105 pg/mL; ⑥ 15.845 μg/L; ⑬ 9.89 mmol/L	⑤⑥⑬	Shock	*OR*	7
Wang Yajuan 2022 (33)	China	89	Case-control	Early-onset sepsis; neonatal necrotizing enterocolitis; concomitant meningitis	Not reported	⑩⑬㊱㊲	Mortality	*OR*	9
Wang Wenhua 2015 (34)	China	100	Case-control	Asphyxia; bleeding	Not reported	⑱	Composite	*OR*	7
Thatrimontrichai 2020 (35)	Thailand	257	Case-control	Complete adjusted covariates were unavailable; the full regression model was not reported in publicly accessible materials, and only the adjusted HR was reported in the abstract	Not reported	㊳	Mortality	*HR*	8
Peng Xiaorui 2025 (36)	China	98	Case-control	Jaundice; body temperature ≥37.5 °C	㊴ 48.995%; ㊵ 46.43%; ㊶ 45.655%	㊴㊵㊶	Composite	*OR*	7

*OR*, odds ratio; *HR*, hazard ratio; NOS, Newcastle–Ottawa Scale; PCT, procalcitonin; MODS, multiple organ dysfunction syndrome; PCIS, Pediatric Critical Illness Score; AST, aspartate aminotransferase; LDH, lactate dehydrogenase; Hb, hemoglobin; ALT, alanine aminotransferase; PT, prothrombin time; INR, international normalized ratio; TT, thrombin time; SNAP-II, Score for Neonatal Acute Physiology II; CPAP, continuous positive airway pressure; PICC, peripherally inserted central catheter. ① RANTES, regulated upon activation, normal T-cell expressed and secreted chemokine; ② CXCL12, C-X-C motif chemokine ligand 12; ③ CXCR4, C-X-C chemokine receptor 4; ④ maternal group B streptococcal infection; ⑤ elevated interleukin-6; ⑥ elevated procalcitonin; ⑦ elevated C-reactive protein; ⑧ RIP3, receptor-interacting protein 3; ⑨ sTREM-1, soluble triggering receptor expressed on myeloid cells 1; ⑩ thrombocytopenia; ⑪ leukopenia; ⑫ base excess < −20 mEq/L; ⑬ elevated lactate; ⑭ hyperglycemia; ⑮ Gram-positive bacterial infection versus fungal infection; ⑯ Gram-positive bacterial infection versus Gram-negative bacterial infection; ⑰ fungal infection versus Gram-negative bacterial infection; ⑱ Gram-negative bacterial infection; ⑲ neutropenia; ⑳ monocyte distribution width; ㉑ neutrophil-to-monocyte ratio; ㉒ decreased albumin; ㉓ platelet count at 24–72 h; ㉔ LL-37, human antimicrobial peptide LL-37; ㉕ MCP-1, monocyte chemoattractant protein 1; ㉖ IL-10, interleukin-10; ㉗ HGB, hemoglobin; ㉘ UREA, urea; ㉙ AIR, serum albumin to international normalized ratio (INR) ratio; ㉚ hypocalcemia; ㉛ hyperphosphatemia; ㉜ pH <7.3; ㉝ IL-8, interleukin-8; ㉞ miR-21, microRNA-21; ㉟ abnormal absolute neutrophil count; ㊱ Listeria infection; ㊲ group B Streptococcus infection; ㊳ Gram-negative bacilli infection; ㊴ CD4+ T lymphocytes; ㊵ Treg, regulatory T cells; ㊶ Treg/CD4 + ratio; ㊷LAR, lactate dehydrogenase-to-albumin ratio.

### 3.3 Meta-analysis results

#### 3.3.1 Overall findings

The 26 studies evaluated risk factors for poor prognosis in neonatal sepsis. Factors that could be quantitatively pooled included elevated interleukin-6, elevated procalcitonin, thrombocytopenia, elevated lactate, elevated C-reactive protein, decreased albumin, Gram-negative bacterial infection, and leukopenia.

#### 3.3.2 Elevated interleukin-6

Four studies provided data suitable for pooling (13, 16, 29, 32). Mild heterogeneity was observed (*I^2^* = 32%, *p* = 0.220), and a fixed-effects model was used. Elevated serum interleukin-6 was associated with an increased risk of poor prognosis in neonatal sepsis (*OR* = 1.97, 95% *CI* 1.42–2.75, *p* < 0.001; Figure 2).

**Figure 2:**
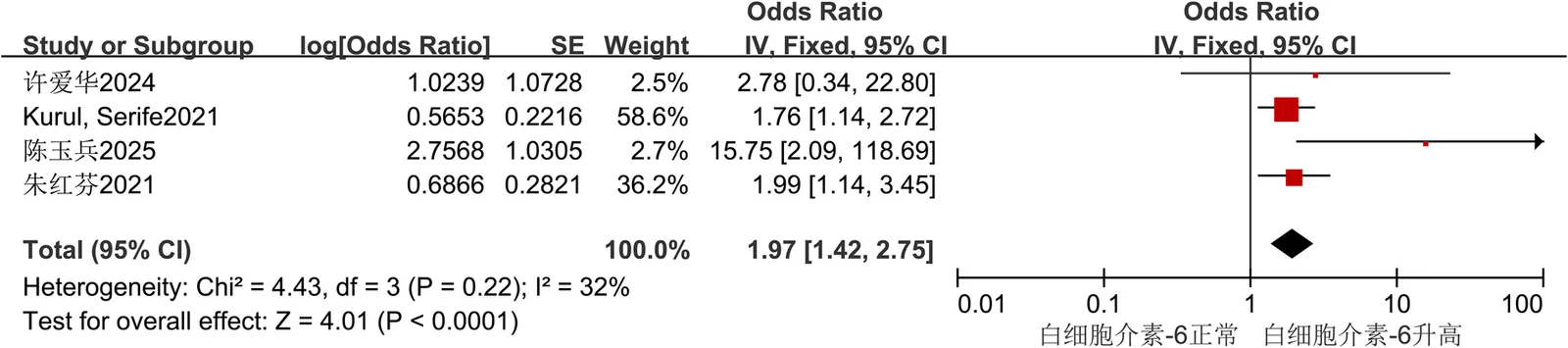
Forest plot of the association between elevated interleukin-6 and poor prognosis in neonatal sepsis.

#### 3.3 Elevated procalcitonin

Three studies provided data suitable for pooling (13, 16, 32). No heterogeneity was detected (*I^2^* = 0%, *p* = 0.400), and a fixed-effects model was used. Elevated procalcitonin was associated with an increased risk of poor prognosis (*OR* = 1.59, 95% *CI* 1.36–1.86, *p* < 0.001; Figure 3).

**Figure 3:**
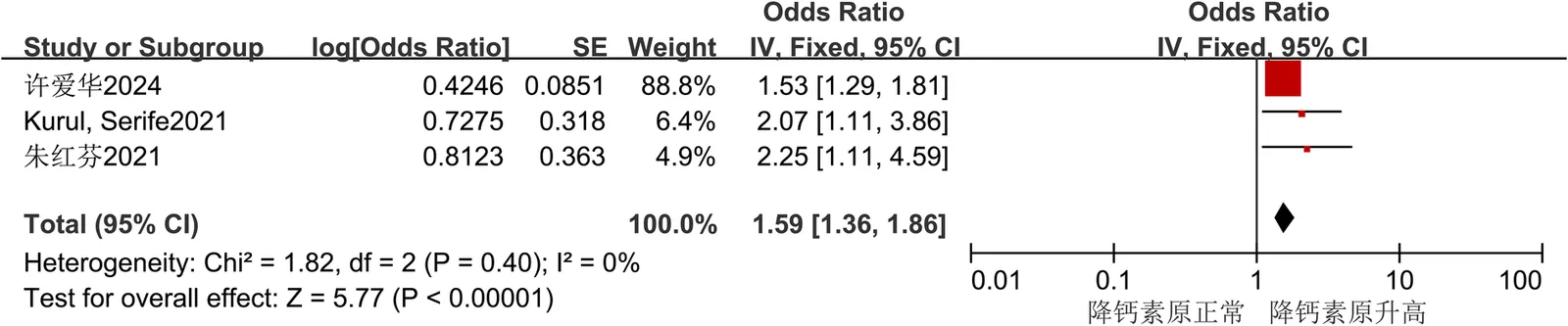
Forest plot of the association between elevated procalcitonin and poor prognosis in neonatal sepsis.

#### 3.3.4 Thrombocytopenia

Six studies provided data suitable for pooling (15, 17, 23, 24, 31, 33). Heterogeneity was high (*I^2^* = 85%, *p* < 0.001), and a random-effects model was used. Thrombocytopenia was associated with an increased risk of poor prognosis (*OR* = 2.47, 95% *CI* 1.15–5.29, *p* = 0.020; Figure 4).

**Figure 4:**
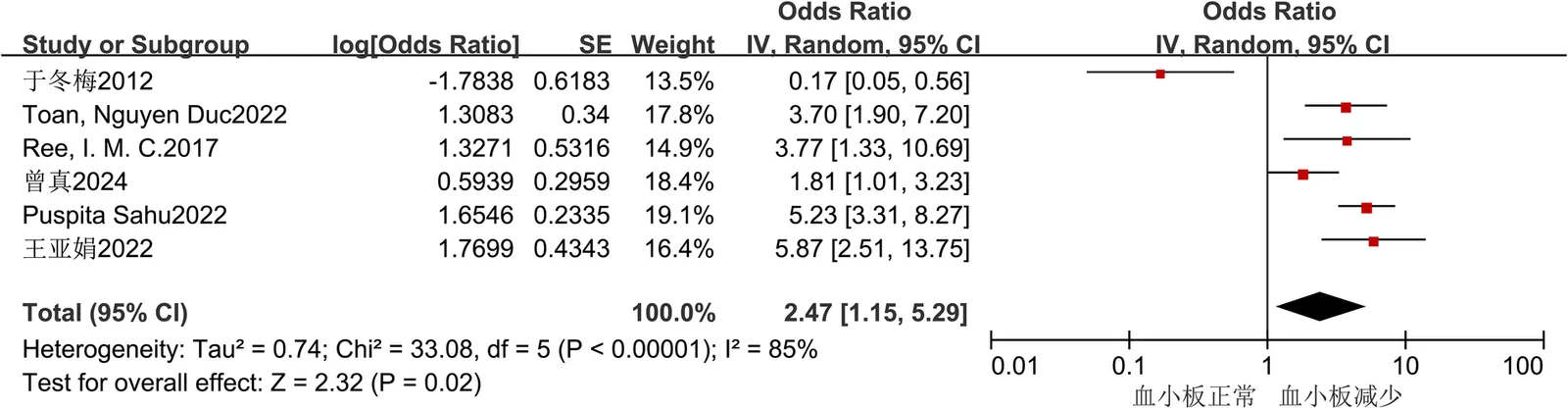
Forest plot of the association between thrombocytopenia and poor prognosis in neonatal sepsis.

#### 3.3.5 Elevated C-reactive protein

Two studies provided data suitable for pooling (13, 31). No heterogeneity was detected (*I^2^* = 0%, *p* = 0.860), and a fixed-effects model was used. Elevated C-reactive protein was associated with an increased risk of poor prognosis (*OR* = 2.04, 95% *CI* 1.38–3.01, *p* = 0.003; Figure 5).

**Figure 5:**
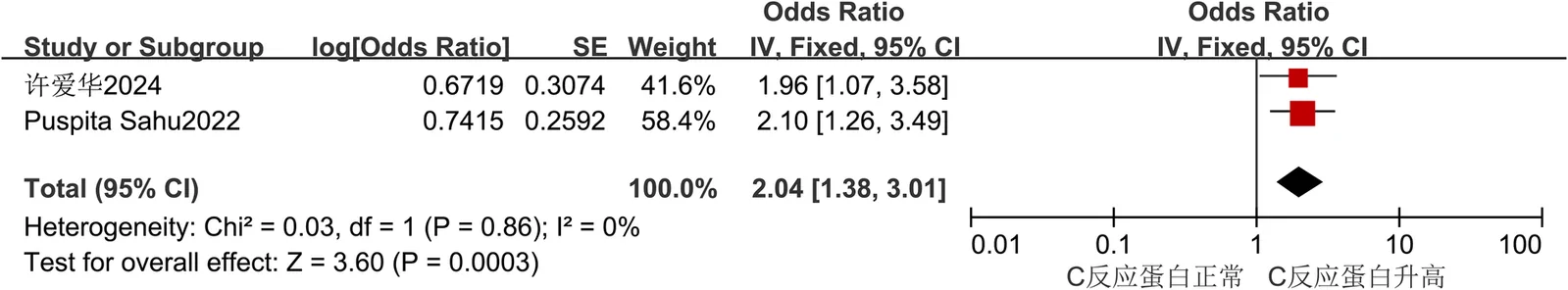
Forest plot of the association between elevated C-reactive protein and poor prognosis in neonatal sepsis.

#### 3.3.6 Leukopenia

Three studies provided data suitable for pooling (17, 24, 31). Heterogeneity was high (*I^2^* = 69%, *p* = 0.040), and a random-effects model was used. Leukopenia was associated with an increased risk of poor prognosis (*OR* = 2.90, 95% *CI* 1.52–5.56, *p* = 0.001; Figure 6).

**Figure 6:**
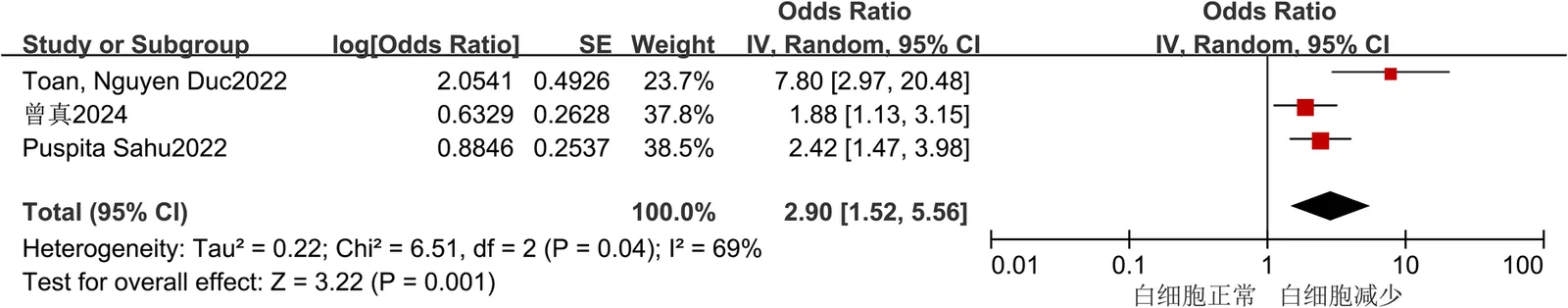
Forest plot of the association between leukopenia and poor prognosis in neonatal sepsis.

#### 3.3.7 Elevated lactate

Three studies provided data suitable for pooling (17, 32, 33). No heterogeneity was observed (*I^2^* = 0%, *p* = 0.450),and a fixed-effects model was used. Elevated lactate was associated with an increased risk of poor prognosis(*OR* = 2.18, 95% *CI* 1.41–3.37, *p* = 0.005; Figure 7).

**Figure 7:**
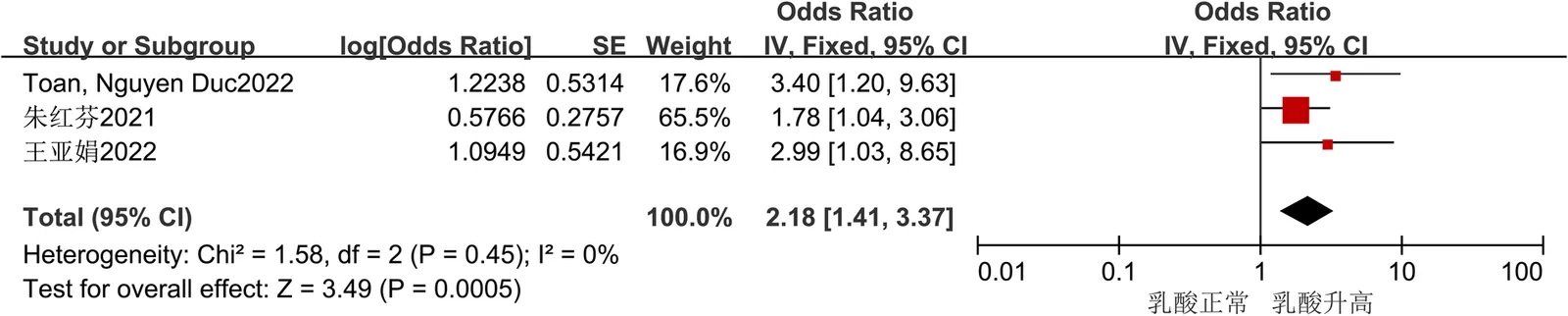
Forest plot of the association between elevated lactate and poor prognosis in neonatal sepsis.

#### 3.3.8 Gram-negative bacterial infection

Two studies provided data suitable for pooling (19, 34). Heterogeneity was low (*I^2^* = 36%, *p* = 0.210), and a fixed-effects model was used. Gram-negative bacterial infection was a potential risk factor for poor prognosis (*OR* = 5.37, 95% *CI* 1.84–15.67, *p* = 0.002; Figure 8).

**Figure 8:**
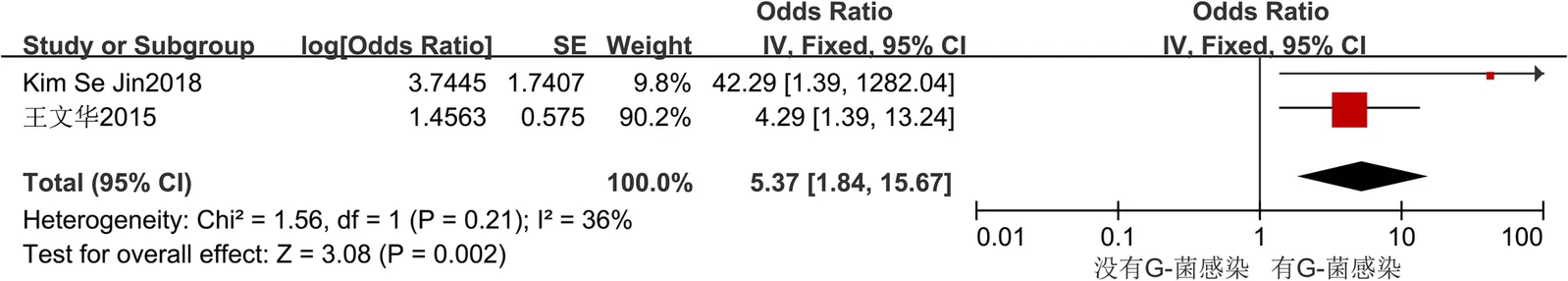
Forest plot of the association between gram-negative bacterial infection and poor prognosis in neonatal sepsis.

#### 3.3.9 Decreased albumin

Two studies provided data suitable for pooling (22, 28). No heterogeneity was detected (*I^2^* = 0%, *p* = 0.360), and a fixed-effects model was used. Decreased albumin was a potential risk factor for poor prognosis (*OR* = 5.77, 95% *CI* 2.38–14.01, *p* = 0.001; Figure 9).

**Figure 9:**
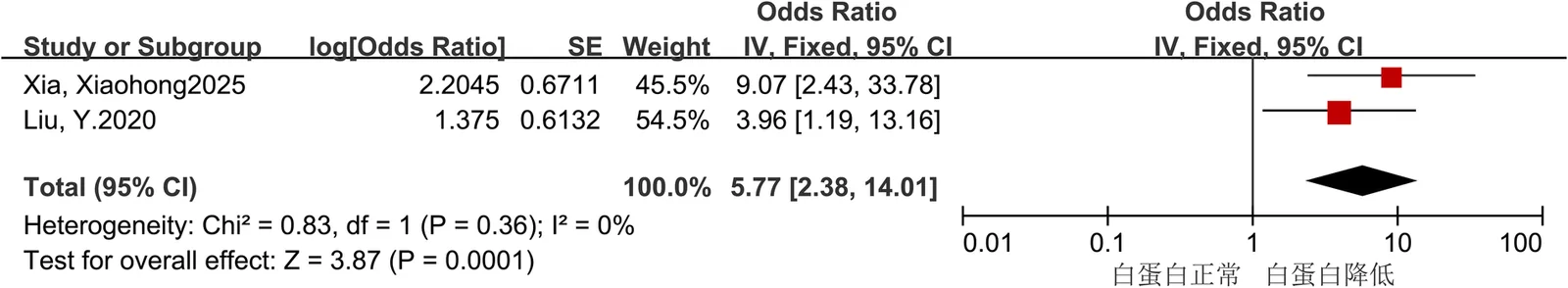
Forest plot of the association between decreased albumin and poor prognosis in neonatal sepsis.

#### 3.3.10 Other factors

Other reported factors included RANTES, CXCL12, CXCR4, maternal group B streptococcal infection, RIP3, sTREM-1, base excess < -20 mEq/L, hyperglycemia, pathogen comparisons, neutropenia, monocyte distribution width, neutrophil-to-monocyte ratio, LAR, platelet count at 24–72 h, LL-37, MCP-1, IL-10, HGB, urea, ALR, hypocalcemia, hyperphosphatemia > 2.6 mmol/L, pH < 7.3, IL-8, miR-21, abnormal absolute neutrophil count, Listeria infection, group B Streptococcus, Gram-negative bacilli infection, CD4+, Treg, and Treg/CD4 + . Each factor was reported by only one study, so effect estimates were not pooled; the original estimates and 95% CIs are summarized in Table 2.

**Table 2:** Other factors associated with poor prognosis in neonatal sepsis.

First author	Factor	*OR*/*HR*	95% *CI*	*p*-value
Jia Panqiang 2025 (11)	RANTES	2.323	1.303–4.142	*p* < 0.001
Jia Panqiang 2026 (11)	CXCL12	2.079	1.242–3.482	*p* = 0.010
Jia Panqiang 2025 (11)	CXCR4	1.938	1.216–3.091	*p* = 0.020
Fan Lili 2023 (12)	Maternal group B streptococcal infection	1.596	1.290–2.372	*p* < 0.050
Hu Yurun 2025 (14)	RIP3	1.445	1.160–1.800	*p* = 0.001
Hu Yurun 2025 (14)	sTREM-1	1.605	1.187–2.170	*p* = 0.002
Toan, Nguyen Duc 2022 (17)	Base excess < -20 mEq/L	3.600	1.300–9.800	*p* = 0.012
Toan, Nguyen Duc 2022 (17)	Hyperglycemia	2.600	1.100–6.100	*p* = 0.021
Levit, O. 2014 (18)	Gram-positive bacteria vs. fungi	0.280	0.090–0.830	*p* = 0.020
Levit, O. 2014 (18)	Gram-positive vs. Gram-negative bacteria	0.160	0.070–0.340	*p* < 0.001
Levit, O. 2014 (18)	Fungi vs. Gram-negative bacteria	0.560	0.200–1.540	*p* = 0.260
Kim Se Jin 2018 (19)	Neutropenia	46.057	1.302–1628.646	*p* = 0.035
Wang, J. 2025 (20)	Monocyte distribution width	3.020	2.020–4.040	*p* < 0.001
Xia Xiaohong 2022 (21)	Neutrophil-to-monocyte ratio	7.519	2.304–24.390	*p* = 0.001
XIA 2025 (22)	LAR	11.236	3.311–38.462	*P* < 0.001
Zhan Yuanli 2021 (25)	Platelet count at 24–72 h	1.031	1.000–1.064	*p* = 0.051
Zhang Ting 2023 (26)	LL-37	6.150	2.469–15.317	*p* < 0.005
Zhang Ting 2023 (26)	MCP-1	5.063	1.298–19.745	*p* < 0.001
Zhang Ting 2023 (26)	IL-10	7.749	3.622–16.579	*p* < 0.005
Xiangwen Tu 2025 (27)	HGB	3.236	1.280–8.197	*p* = 0.013
Xiangwen Tu 2025 (27)	UREA	8.044	1.018–63.538	*p* = 0.048
Xiangwen Tu 2025 (27)	ALR	9.091	3.106–26.316	*p* < 0.001
Liu, Y. 2020 (28)	hypocalcemia	4.272	1.267–14.407	*p* = 0.019
Liu, Y. 2020 (28)	hyperphosphatemia > 2.6 mmol/L	5.822	1.477–22.948	*p* = 0.012
Liu, Y. 2020 (28)	pH < 7.3	6.472	1.828–22.914	*p* = 0.004
Chen Yubing 2025 (29)	IL-8	8.960	2.340–34.320	*p* = 0.001
Rabab F. Salim 2020 (30)	miR-21	0.422	0.200–0.891	*p* = 0.024
Puspita Sahu 2022 (31)	Abnormal absolute neutrophil count	2.108	1.451–3.062	*p* < 0.001
Wang Yajuan 2022 (33)	Listeria infection	4.865	3.882–6.097	*p* < 0.001
Wang Yajuan 2022 (33)	Group B Streptococcus	7.182	4.026–12.811	*p* < 0.001
Anucha Thatrimontrichai 2020 (35)	Gram-negative bacilli infection	6.670	3.280–13.570	*p* < 0.001
Peng Xiaorui 2025 (36)	CD4+	1.930	1.193–5.247	*p* = 0.029
Peng Xiaorui 2025 (36)	Treg	2.513	1.231–5.439	*p* = 0.022
Peng Xiaorui 2025 (36)	Treg/CD4+	2.241	1.039–4.943	*p* = 0.039

### 3.4 Sensitivity analysis

Sensitivity analyses and exploration of heterogeneity were performed for thrombocytopenia, leukopenia, and elevated lactate because I^2^ > 50%. A leave-one-out approach was used, in which each study was sequentially excluded and the meta-analysis was repeated. Comparison of the results showed that thrombocytopenia and leukopenia remained risk factors. Yu Dongmei 2012 (15) appeared to be a source of heterogeneity for thrombocytopenia; after its exclusion, *I^2^* decreased to 56% and the pooled *OR* was 3.72. Toan, Nguyen Duc 2022 (17) appeared to be a source of heterogeneity for leukopenia; after exclusion, *I^2^* decreased to 0% and the pooled *OR* was 2.15. (Table 3).

**Table 3:** Sensitivity analyses.

Factor	Study excluded	*I^2^* before (%)	*OR* before (95% *CI*)	*p* before	*I^2^* after (%)	*OR* after (95% *CI*)	*p* after
Thrombocytopenia	Yu Dongmei 2012 (15)	85	2.47 (1.15–5.29)	0.020	56	3.72 (2.35–5.88)	<0.001
Leukopenia	Toan, Nguyen Duc 2022 (17)	69	2.90 (1.52–5.56)	0.001	0	2.15 (1.50–3.07)	<0.001

### 3.5 Publication bias

Because fewer than 10 studies were included in each pooled analysis, formal assessment of publication bias was not performed.

## 4 Discussion

This systematic review and meta-analysis synthesized current evidence on prognostic factors in neonatal sepsis. Elevated interleukin-6, elevated procalcitonin, thrombocytopenia, elevated lactate, elevated C-reactive protein, decreased albumin, Gram-negative bacterial infection, and leukopenia were identified as important factors associated with poor prognosis. Their potential clinical implications are discussed below.

### 4.1 Elevated interleukin-6

Elevated IL-6 was associated with an increased risk of poor prognosis (*OR* = 1.97, 95% *CI* 1.42–2.75, *P* < 0.001; *I^2^* = 32%, *P* = 0.220). Zhao et al. (37) reported that IL-6 has prognostic value in neonates with sepsis, consistent with the present findings. IL-6 is a pro-inflammatory cytokine (38). Its concentration may increase within 2 h after disease onset, peak at approximately 6 h, and decline within 24 h (39). IL-6 levels also increase with increasing disease severity (40). These findings support the clinical relevance of IL-6 in sepsis (41).

### 4.2 Elevated C-reactive protein

Elevated CRP was associated with an increased risk of poor prognosis (*OR* = 2.04, 95% *CI* 1.38–3.01, *P* = 0.003; *I^2^* = 0%, *P* = 0.860). Piccioni et al. (42) noted that CRP is a sepsis biomarker with value for prognostic assessment, consistent with the present findings. CRP is an acute-phase protein (43). Mechanistically, CRP activates the C1q molecule in the complement pathway and thereby promotes opsonization of pathogens (44). Following an inflammatory stimulus, CRP rises rapidly within 6–8 h, reaches a peak at approximately 48 h, and returns toward baseline as inflammation resolves (45). Therefore, clinical measurement of CRP may facilitate recognition of sepsis and support timely intervention when necessary (46).

### 4.3 Elevated procalcitonin

Elevated PCT was associated with an increased risk of poor prognosis (*OR* = 1.59, 95% *CI* 1.36–1.86, *P* < 0.001; *I^2^* = 0%, *P* = 0.40). Xu et al. (47) reported that changes in PCT are useful for the diagnosis and prognostic assessment of sepsis, which is consistent with our findings. PCT is a biomarker of the systemic inflammatory response (48). In neonatal sepsis, PCT begins to increase approximately 4 h after onset and reaches a peak within 6–8 h (49). Elevated PCT may indicate a higher risk of severe outcomes, supporting targeted interventions and closer monitoring of high-risk pediatric patients (50).

### 4.4 Thrombocytopenia

Thrombocytopenia was associated with an increased risk of poor prognosis (*OR* = 2.47, 95% *CI* 1.15–5.29, *P* = 0.020; *I^2^* = 85%, *P* < 0.001). Yu Dongmei 2012 (15) appeared to be a potential source of heterogeneity. After exclusion of this study, *I^2^* decreased to 56% (*P* = 0.060), and the association remained significant (*OR* = 3.72, 95% *CI* 2.35–5.88, *P* < 0.001). One possible explanation is that thrombocytopenia appeared to be a protective factor for poor prognosis in the study by Yu Dongmei 2012 (15), whereas it was identified as a risk factor in the other included studies. Previous research has shown that severe neonatal thrombocytopenia is associated with poor overall outcomes (51), consistent with our findings. Thrombocytopenia is defined as a platelet count <150 × 10⁹/L (52). Sepsis can cause endothelial injury and activate the reticuloendothelial system, leading to platelet consumption that exceeds platelet production and ultimately resulting in thrombocytopenia (53). Clinically, neonates at risk of sepsis should therefore be identified promptly and treatment initiated without delay (54).

### 4.5 Elevated lactate

Elevated lactate was associated with an increased risk of poor prognosis (*OR* = 2.18, 95% *CI* 1.41–3.37, *P* = 0.005; *I^2^* = 0%, *P* = 0.450). Kislal et al. (55) reported that lactate may be useful for prognostic assessment in neonatal sepsis, consistent with the present findings. Lactate is a metabolic by-product of the host and intestinal microbiota (56). In neonatal sepsis, inadequate tissue perfusion may enhance anaerobic glycolysis and increase lactate production (57). Accordingly, timely assessment of lactate may facilitate recognition of disease progression and support early clinical management (58).

### 4.6 Decreased albumin

Decreased ALB was associated with poor prognosis (*OR* = 5.77, 95% *CI* 2.38–14.01, *P* = 0.001; *I^2^* = 0%, *P* = 0.360). Wang Ying (59) reported that lower ALB levels were associated with poorer outcomes in neonatal sepsis, consistent with our findings. Hypoalbuminemia reflects a reduction in circulating protein levels (60). During sepsis, the release of inflammatory mediators may disrupt the balance between pro-inflammatory and anti-inflammatory responses and promote excessive free-radical generation. Reduced ALB may weaken the body's capacity to scavenge free radicals and thereby contribute to disease progression (61). Early identification of neonates at risk may therefore help optimize the timing of empirical antibiotic therapy and subsequent management of sepsis (62).

### 4.7 Gram-negative bacterial infection

Gram-negative bacterial infection was associated with poor prognosis (*OR* = 5.37, 95% *CI* 1.84–15.67, *P* = 0.002; *I^2^* = 36%, *P* = 0.210). Coggins et al. (63) reported that sepsis caused by Gram-negative pathogens is associated with substantial mortality, consistent with our findings. The immature neonatal immune system may increase susceptibility to sepsis (64). Wu et al. (65) further reported that pathogen-induced sequential organ dysfunction may progress to interacting multiple-organ failure, aggravating disease severity, with mortality increasing as the number of failing organs increases. Early identification of causative pathogens and clinical manifestations is therefore important for timely diagnosis and intervention (66)^.^

### 4.8 Leukopenia

Leukopenia was associated with an increased risk of poor prognosis (*OR* = 2.90, 95% *CI* 1.52–5.56, *P* = 0.001; *I^2^* = 69%, *P* = 0.001). Toan 2022 (17) appeared to be a potential source of heterogeneity. After exclusion of this study, heterogeneity was eliminated (*I^2^* = 0%, *P* = 0.490), and the association remained significant (*OR* = 2.15, 95% *CI* 1.50–3.07, *P* < 0.001). A possible explanation is that the study by Toan et al. (17) had an NOS score of 6, whereas the other two studies had NOS scores of 7, suggesting potential differences in study design, methodological quality, or control of bias. Zhang Weixin et al. (67) reported that leukopenia may reflect disease severity and prognosis in neonatal sepsis, consistent with our findings. Leukopenia is defined as a peripheral white blood cell count below the neonatal reference range (68). Interactions between leukocytes and platelets during sepsis may contribute to reductions in both cell populations (69). Inflammatory mediators and bacterial toxins may also directly damage leukocytes, thereby reducing their number (70). Given the limited evidence on leukopenia and poor prognosis in neonatal sepsis, further studies are warranted to clarify its prognostic value and provide a stronger evidence base for clinical practice.

### 4.9 Other factors

The included studies also reported RANTES, CXCL12, CXCR4, maternal group B streptococcal infection, RIP3, sTREM-1, base excess < -20 mEq/L, hyperglycemia, pathogen comparisons, neutropenia, monocyte distribution width, neutrophil-to-monocyte ratio, LAR, platelet count at 24–72 h, LL-37, MCP-1, IL-10, HGB, urea, ALR, hypocalcemia, hyperphosphatemia > 2.6 mmol/L, pH < 7.3, IL-8, miR-21, abnormal absolute neutrophil count, and Listeria infection. Each was reported in only one study; consequently, no pooled analysis was performed and these factors were not discussed in detail.

### 4.10 Strengths and limitations

All included studies used multivariable analyses, which supports the overall robustness of the findings; however, several limitations should be acknowledged. First, relatively few studies were conducted outside China, which may have introduced geographic bias. Second, cut-off values varied across studies, preventing the establishment of uniform thresholds for some prognostic factors. Third, differences in measurement instruments and related laboratory procedures may have introduced measurement bias. Fourth, because publication bias and subgroup analyses were not performed, the pooled effect estimates may have been overestimated. Fifth, although the review included 19,673 neonates, only 26 studies met the eligibility criteria; therefore, the findings require confirmation in additional high-quality studies. Sixth, all types of neonatal sepsis were analyzed together without subgrouping by time of onset because of sample-size limitations. Thus, although the identified biomarkers may have diagnostic or prognostic potential in neonatal sepsis, their ability to distinguish early-onset sepsis (EOS) from late-onset sepsis (LOS) requires further investigation. Seventh, several pooled estimates were based on only two studies, and even the largest pooled analysis included only 6 of the 26 studies, making the pooled estimates susceptible to the influence of individual studies; these findings should therefore be considered preliminary. Eighth, because the original studies provided limited information on sampling time points, we were unable to account directly for differences in specimen collection timing. Although sensitivity analyses were used to assess the overall stability of the results, the lack of direct adjustment for sampling time may still affect the robustness of the pooled estimates. Ninth, although elevated blood lactate was significantly associated with poor prognosis, this finding should be interpreted cautiously because lactate is often measured from capillary blood samples in clinical practice, which may lead to spuriously elevated values. Tenth, outcome definitions were not fully consistent across the included studies: most studies used mortality as the primary outcome, whereas some used composite outcomes, which may have affected the pooled effect estimates. Finally, several prognostic factors were reported by only one study and therefore could not be quantitatively pooled.

## 5 Conclusion

In conclusion, elevated IL-6, elevated PCT, thrombocytopenia, elevated lactate, elevated CRP, decreased ALB, Gram-negative bacterial infection, and leukopenia were identified as independent risk factors for poor prognosis in neonatal sepsis. Clinicians should pay particular attention to neonates with these characteristics. Further prospective, multicenter studies are needed to validate these findings, investigate interactions among risk factors, and evaluate the clinical utility of multivariable prognostic prediction models. In addition, factors that could not be quantitatively pooled may provide directions for future diagnostic and therapeutic research.

## Data Availability

All data analyzed in this meta-analysis were extracted from published studies. The synthesized results of this analysis are available within the manuscript and supplementary materials.
